# Synthesis and Spectrum of Biological Activities of Novel *N*-arylcinnamamides

**DOI:** 10.3390/ijms19082318

**Published:** 2018-08-07

**Authors:** Sarka Pospisilova, Jiri Kos, Hana Michnova, Iva Kapustikova, Tomas Strharsky, Michal Oravec, Agnes M. Moricz, Jozsef Bakonyi, Tereza Kauerova, Peter Kollar, Alois Cizek, Josef Jampilek

**Affiliations:** 1 Department of Pharmaceutical Chemistry, Faculty of Pharmacy, Comenius University, Odbojarov 10, 83232 Bratislava, Slovakia; sharka.pospisilova@gmail.com (S.P.); michnova.hana@gmail.com (H.M.); kapustikova@fpharm.uniba.sk (I.K.); strharsky2@uniba.sk (T.S.); josef.jampilek@gmail.com (J.J.); 2 Department of Infectious Diseases and Microbiology, Faculty of Veterinary Medicine, University of Veterinary and Pharmaceutical Sciences, Palackeho 1, 61242 Brno, Czech Republic; cizeka@vfu.cz; 3 Global Change Research Institute CAS, Belidla 986/4a, 60300 Brno, Czech Republic; oravec.m@czechglobe.cz; 4 Plant Protection Institute, Centre for Agricultural Research, Hungarian Academy of Sciences, Herman Otto Str. 15, 1022 Budapest, Hungary; moricz.agnes@agrar.mta.hu (A.M.M.); bakonyi.jozsef@agrar.mta.hu (J.B.); 5 Department of Human Pharmacology and Toxicology, Faculty of Pharmacy, University of Veterinary and Pharmaceutical Sciences, Palackeho 1, 61242 Brno, Czech Republic; tereza.kauerova@gmail.com (T.K.); kollarp@vfu.cz (P.K.)

**Keywords:** cinnamamides, antistaphylococcal activity, time-kill assay, biofilm, antitubercular activity, MTT assay, antifungal activity, PET inhibition, toxicity, structure–activity relationship

## Abstract

A series of sixteen ring-substituted *N*-arylcinnamamides was prepared and characterized. Primary in vitro screening of all the synthesized compounds was performed against *Staphylococcus aureus*, three methicillin-resistant *S. aureus* strains, *Mycobacterium tuberculosis* H37Ra, *Fusarium avenaceum*, and *Bipolaris sorokiniana*. Several of the tested compounds showed antistaphylococcal, antitubercular, and antifungal activities comparable with or higher than those of ampicillin, isoniazid, and benomyl. (2*E*)-*N*-[3,5-bis(trifluoromethyl)phenyl]-3-phenylprop-2-enamide and (2*E*)-3-phenyl-*N*-[3-(trifluoromethyl)phenyl]prop-2-enamide showed the highest activities (MICs = 22.27 and 27.47 µM, respectively) against all four staphylococcal strains and against *M. tuberculosis*. These compounds showed an activity against biofilm formation of *S. aureus* ATCC 29213 in concentrations close to MICs and an ability to increase the activity of clinically used antibiotics with different mechanisms of action (vancomycin, ciprofloxacin, and tetracycline). In time-kill studies, a decrease of CFU/mL of >99% after 8 h from the beginning of incubation was observed. (2*E*)-*N*-(3,5-Dichlorophenyl)- and (2*E*)-*N*-(3,4-dichlorophenyl)-3-phenylprop-2-enamide had a MIC = 27.38 µM against *M. tuberculosis*, while a significant decrease (22.65%) of mycobacterial cell metabolism determined by the MTT assay was observed for the 3,5-dichlorophenyl derivative. (2*E*)-*N*-(3-Fluorophenyl)- and (2*E*)-*N*-(3-methylphenyl)-3-phenylprop-2-enamide exhibited MICs = 16.58 and 33.71 µM, respectively, against *B*. *sorokiniana*. The screening of the cytotoxicity of the most effective antimicrobial compounds was performed using THP-1 cells, and these chosen compounds did not shown any significant lethal effect. The compounds were also evaluated for their activity related to the inhibition of photosynthetic electron transport (PET) in spinach (*Spinacia oleracea* L.) chloroplasts. (2*E*)-*N*-(3,5-dichlorophenyl)-3-phenylprop-2-enamide (IC_50_ = 5.1 µM) was the most active PET inhibitor. Compounds with fungicide potency did not show any in vivo toxicity against *Nicotiana tabacum* var. Samsun. The structure–activity relationships are discussed.

## 1. Introduction

Cinnamic acids and other hydroxy- or phenyl-substituted derivatives of cinnamic acids have been widely investigated by scientists due to their significant and varied biological effects. Cinnamic acids occur naturally in all plants [1]. They are formed in the biochemical pathway providing phenyl-propanoids, coumarins, lignans, isoflavonoids, flavonoids, stilbenes, aurones, anthocyanins, spermidines, and tannins [2]. The spectrum of their biological activities include anti-inflammatory, antioxidant, hepatoprotective, antidiabetic, antidepressant/anxiolytic, antifungal, antibacterial, antiviral, and anticancer effects [3,4,5,6,7,8,9,10,11,12,13,14,15,16]. Derivatives of cinnamic acids are used as agriculture fungicides as well [17].

The adaptation of microorganisms to external influences and, thus, the development of their resistance against antimicrobial agents is not a surprise; unfortunately, this process is faster and faster. Thus, the emerging resistance of microbial pathogens to clinically used drugs, including second- and third-choice drugs, and the development of cross-resistant or multidrug-resistant strains are alarming. Microbial pathogens have developed a number of mechanisms to adapt to the effects of the environment. In addition, the increase in the number of infections and the occurrence of new, especially opportunistic species are also caused by the general immunosuppression of patients, and this fact makes these diseases extremely serious. Since the 1990s, only an inconsiderable number of really new drugs for systemic administration have been marketed for the treatment of infections, although the discovery of new molecules has been a priority [18].

Thus, in the light of the above mentioned facts, new simple anilides of cinnamic acid were designed as antimicrobial multitarget agents, synthesized using a modern microwave-assisted method and screened against a battery of bacterial/mycobacterial and fungal pathogens. These compounds were designed based on the experience with naphthalenecarboxamides—simple molecules with a number of biological activities, and in fact, the new ring-substituted (2*E*)-*N*-phenyl-3-phenylprop-2-enamides can be considered as open analogues of recently described naphthalene-2-carboxanilides [19,20].

As an amide moiety is able to affect photosystem II (PS II) by reversible binding [21], resulting in the interruption of the photosynthetic electron transport (PET) [22,23,24], and can be found in many herbicides acting as photosynthesis inhibitors [25,26,27,28,29,30], these *N*-arylcinnamamides were additionally tested on the inhibition of PET in spinach (*Spinacia oleracea* L.) chloroplasts using the Hill reaction. The idea of this screening is based on the fact that both drugs and pesticides are designed to target particular biological functions. These functions/effects may overlap at the molecular level, which causes a considerable structural similarity between drugs and pesticides. Since different classes of herbicides are able to bind to different mammalian cellular receptors, the majority of pharmaceutical companies have pesticide divisions, and developed biologically active agents are investigated as both pesticides and drugs. Previously, several successful pesticides became pharmaceuticals and vice versa [31,32,33,34,35].

## 2. Results and Discussion

### 2.1. Chemistry and Physicochemical Properties

**Scheme 1:**
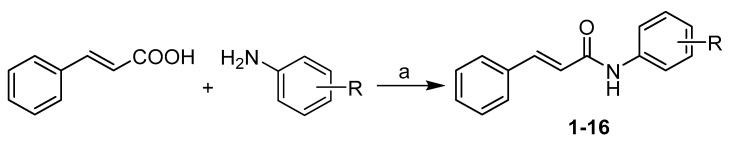
Synthesis of (2*E*)-*N*-aryl-3-phenylprop-2-enamides **1**–**16**. Reagents and conditions: (a) PCl_3_, chlorobenzene, and MW.

All the studied compounds **1**–**16** were prepared according to Scheme 1. The carboxyl group of starting cinnamic acid was activated with phosphorus trichloride. In the reaction with an appropriate ring-substituted aniline, the generated acyl chloride subsequently gave the final amide in dry chlorobenzene via microwave-assisted synthesis. All the compounds were recrystallized from ethanol.

Many different molecular parameters/descriptors are used to determine structure-activity relationships (SAR). Lipophilicity and electronic properties are among the most frequent ones. Hammett’s σ parameters were used for the description of electronic properties. They were calculated for the whole substituted anilide ring using ACD/Percepta ver. 2012 (Advanced Chemistry Development Inc., Toronto, ON, Canada, 2012), see Table 1. The lipophilicity of the studied compounds was predicted as log *P* using ACD/Percepta software and Clog *P* using ChemBioDraw Ultra 13.0 (CambridgeSoft, PerkinElmer Inc., Cambridge, MA, USA). Log *P* is the logarithm of the partition coefficient for *n*-octanol/water. Clog *P* is the logarithm of *n*-octanol/water partition coefficient based on the established chemical interactions. In addition, the lipophilicity of studied compounds **1**–**16** was investigated by means of reversed-phase high-performance liquid chromatography (RP-HPLC) determination of capacity factors *k* with the subsequent calculation of log *k* [36]. The analysis was made under isocratic conditions with methanol as an organic modifier in the mobile phase using an end-capped nonpolar C18 stationary RP column. The results are shown in Table 1.

**Figure 1:**
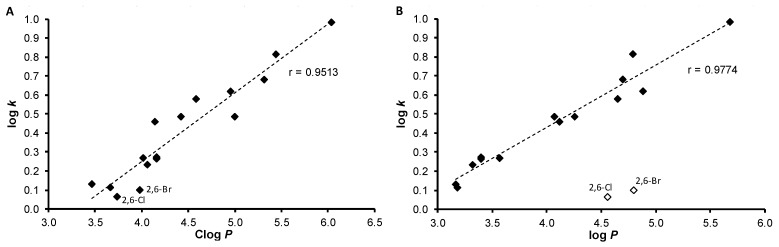
Comparison of experimentally found log *k* values of ring-substituted *N*-arylcinnamamides **1**–**16** with Clog *P* calculated using ChemBioDraw Ultra (**A**) and log *P* calculated using ACD/Percepta (**B**).

The results obtained with the discussed compounds show that the experimentally-determined lipophilicities (log *k*) are in accordance with the calculated Clog *P* values as illustrated in Figure 1A; correlation coefficient *r* = 0.9513, *n* = 16. On the other hand, log *P* values calculated by ACD/Percepta show differences for compounds **9** (2,6-Cl) and **12** (2,6-Br), see Figure 1B. When these two compounds are excluded, *r* = 0.9774 (*n* = 14) is observed. This poor match for 2,6-disubstituted anilides **9** and **12** may be caused by intramolecular interactions that are probably caused by the steric effect of spatially-close moieties, which was not included in prediction by ACD/Percepta. The proximity of the di-*ortho*-substituents to the carboxamide group on the aniline ring leads to the twist of the aniline ring plane towards the carboxamide group, i.e., to the plane of the benzene ring of cinnamic acid. The described process resulted in the planarity violation of the molecule. Otherwise, (2*E*)-*N*-[3,5-bis(trifluoromethyl)phenyl]-3-phenylprop-2-enamide (**13**) is the most lipophilic, while compounds **9**, **12** and *N*-phenylcinnamamide (**1**) are characterized by the lowest lipophilicity. It can be stated that log *k* values specify lipophilicity within the series of the studied compounds.

### 2.2. In Vitro Antibacterial Susceptibility Testing

All the cinnamanilides were tested on their antistaphylococcal activity against three clinical isolates of methicillin-resistant *Staphylococcus aureus* (MRSA) [37,38] and *S. aureus* ATCC 29213 as the reference and quality control strain. Although various derivatives of cinnamic acid were described as promising antibacterial agents [4,5,6,8,9,14,15], the compounds showed only limited activity (MICs > 256 µg/mL), except for (2*E*)-3-phenyl-*N*-[3-(trifluoromethyl)phenyl]prop-2-enamide (**6**) and 3,5-bis(trifluoromethyl)phenyl derivative **13**, see Table 2. As minimum inhibitory concentrations (MICs) of these compounds are the same against the reference and the MRSA strains (27.47 and 22.27 µM, respectively), it can be speculated about the specific effectivity against *Staphylococcus* sp. These compounds were also tested against *Enterococcus faecalis* ATCC 29212 as the reference strain and three isolates from American crows of vanA-carrying vancomycin-resistant *E. faecalis* (VRE) [39] but without any effect in the tested concentrations, which may indicate a specific mechanism of action [37,40]. From Table 2 it is obvious that compounds **6** and **13** exhibited activities comparable with those of the standards. Due to the small number of active compounds, no SAR could be established.

#### 2.2.1. Synergy Effect with Clinically Used Drugs against MRSA

The most effective compounds **6** and **13** were tested for their ability of synergic activity with clinically used antibacterial drugs tetracycline, ciprofloxacin, and vancomycin. These antibiotics have different mechanisms of actions and different mechanisms of resistance to them, thus the prospective synergism could give an idea of the mechanism of action of the cinnamic derivatives. The investigation of synergistic activity was performed according to the methodology [41]. The method of fractional inhibitory concentration (FIC) was used [42]. For all the wells of the microtitration plates that corresponded to a MIC value, the sum of the FICs (ΣFIC) was calculated for each well, using the equation ΣFIC = FIC_A_ + FIC_B_ = (C_A_/MIC_A_) + (C_B_/MIC_B_), where MIC_A_ and MIC_B_ are the MICs of drugs A and B alone, respectively, and C_A_ and C_B_ are the concentrations of the drugs in the combination, respectively [42]. Synergy was defined as a ΣFIC ≤ 0.5; additivity was defined as 0.5 < ΣFIC < 1; indifference was defined as 1 ≤ ΣFIC < 4; and antagonism was defined as ΣFIC ≥ 4 [41]. As the FIC index was evaluated for every single well corresponding to the MIC value, the results are presented as a range. The test was made with all 3 methicillin-resistant isolates, MRSA 63718, SA 3202, and SA 630. The isolates were also resistant to used antibiotics. Note that isolate MRSA SA 630 is susceptible to tetracycline. The results are mentioned in Table 3.

Although the activity of cinnamic acid derivatives is known for a long time, the exact mechanism of action is still unknown. The most reported mechanism of action is interaction with plasmatic membrane. The compounds can cause disruption of the membrane, damage the membrane proteins, etc. [43,44,45,46]. There are also specific targets for cinnamic acid derivatives [46]. Nevertheless, it is possible that the wide spectrum of effects to cells is caused by the primary activity of the compounds, which is membrane destabilization [46].

Both compounds 6 and 13 tested for synergy showed additivity with vancomycin against MRSA SA 630 and SA 3202. A similar effect was reported by Hemaiswarya et al. [47]; compound 13 had synergistic effect with ciprofloxacin against both tested strains. The effect of derivative 13 was also synergistic with tetracycline against MRSA SA 3202. The rest combinations with compound 13 had additive effect. Whereas compound 13 had a potential to increase the activity of all tested antibiotics, which have different mechanisms of actions and to which bacteria develop different resistance mechanisms, it can be expected that compound 13 acts by its own mechanism of action or increases the availability of the antibiotics by interaction with the membrane.

#### 2.2.2. Dynamics of Antibacterial Activity

**Figure 2:**
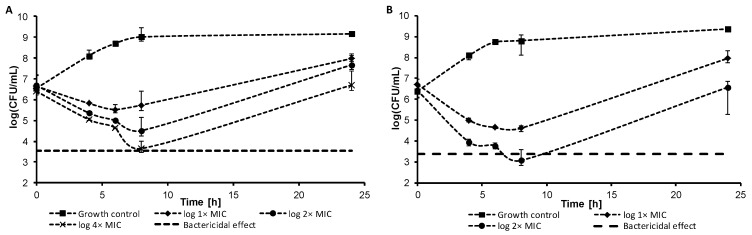
Time-kill curve of compound **6** (**A**) and compound **13** (**B**) against *S. aureus* ATCC 29213.

Within the pre-test subcultivation aliquots on agar, antistaphylococcal-effective compounds **6** and **13** showed bactericidal activity, i.e., minimal bactericidal concentrations were ≤4× MIC. These facts were verified using the time-kill curve assay for testing the bactericidal effect. The dynamics of antibacterial activity was tested against *S. aureus* ATCC 29213 for the most active compounds **6** (Figure 2A) and **13** (Figure 2B). Both compounds showed concentration-dependent activity that was bactericidal in concentration 4× MIC in the case of compound **13** or very close to the bactericidal level for compound **6** after 8 h from the beginning of incubation. The increase of bacterial growth at 24 h could be caused by the selection of resistant mutants, as observed previously [37].

#### 2.2.3. Inhibition of Bofilm Formation

There are many evidences in literature that cinnamic acid derivatives are inhibitors of biofilm formation [47,48,49,50,51]. The most studied derivate is cinnamaldehyde that interacts with quorum sensing system in bacterial biofilms [46,52,53]. Thus, selected compounds were also tested for their ability to inhibit biofilm formation. Compounds **6** and **13** were tested as inhibitors of biofilm formation against *S. aureus* ATCC 29213. MRSA strains were not producers of biofilm.

The activity of compound **6** does not depend on concentration in concentrations above 8 µg/mL; only the highest concentration showed lower inhibition effect. This could be caused the higher lipophilicity of the compound and potential formation of precipitates, which could decrease the antibacterial activity of the compound. The lowest concentration of the compound, which inhibited ≥80% of biofilm formation, was 8 µg/mL, then the activity sharply decreased. Interestingly, on the other hand, concentrations of compound **13** close to MIC against planktonic cells had the lowest inhibition activities against biofilm forming, and the activity increased for sub-MIC values. These conditions could be potentially toxic for planktonic cells, but they can induce biofilm formation [54]. In general, the inhibition activity against biofilm formation was comparable with the activity against planktonic cells. Despite many studies reported a higher resistance of biofilm, there were also studies that proved a similar or only little lower antibiofilm activity of tested compounds compared to planktonic cells. [13,55]. Budzynska et al. [55] described high antibiofilm activity of plant essential oils compared to MICs. De Vita et al. [13] studied the activity of cinnamic acid derivatives against candida biofilm. These compounds had good effect against biofilm formation, and the effective concentrations were lower than 10× MIC. Thus, the high activity of our compounds can be explained due to their structure, based on cinnamic acid.

**Figure 3:**
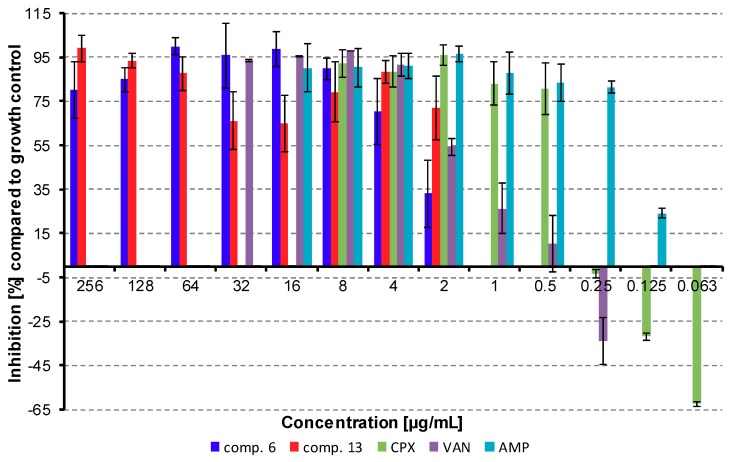
Inhibition of bacterial film formation. (CPX = ciprofloxacin, AMP = ampicillin, VAN = vancomycin).

Ampicillin (16–0.125 µg/mL), vancomycin (32–0.25 µg/mL), and ciprofloxacin (8–0.063 µg/mL) were used as positive controls. Ciprofloxacin and vancomycin caused the induction of biofilm formation in sub-MIC concentrations, which is in line with already published results [56,57]. All the results are shown in Figure 3.

### 2.3. In Vitro Antitubercular Activity

The evaluation of the in vitro antitubercular activity of the compounds was performed against *Mycobacterium tuberculosis* ATCC 25177/H37Ra, see Table 2. In order to reduce risks, a replacement of model pathogens is commonly used in basic laboratory screening. For *M. tuberculosis*, avirulent strain H37Ra is used that has a similar pathology as *M. tuberculosis* strains infecting humans and, thus, represents a good model for testing antitubercular agents [58]. The potency of the compounds was expressed as the MIC that is defined for mycobacteria as 90% or greater (IC_90_) reduction of growth in comparison with the control.

**Figure 4:**
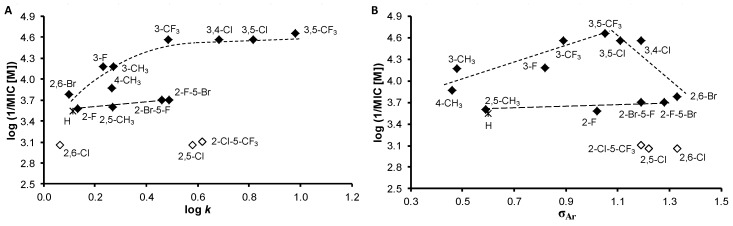
Relationships between in vitro antitubercular activity against *M. tuberculosis* log (1/MIC (M)) and lipophilicity expressed as log *k* (**A**) and electronic Hammett’s σ parameters of ring-substituted anilide ring (**B**) of studied compounds. (Derivatives excluded from SAR are illustrated by empty symbols.).

In comparison with antibacterial activities, the investigated compounds exhibited much higher effect against *M. tuberculosis*. Compounds **13** (R = 3,5-CF_3_), **10** (R = 3,4-Cl), **11** (R = 3,5-Cl), and **6** (R = 3-CF_3_) were the most effective; their activity ranged from 22.27 to 27.47 µM. The dependences of the antitubercular activity of the compounds against *M. tuberculosis* expressed as log (1/MIC (M)) on lipophilicity expressed as log *k* are illustrated in Figure 4A. When inactive compounds **8** (R = 2,5-Cl), **9** (R = 2,6-Cl), and **16** (R = 2-Cl-5-CF_3_) are eliminated from the SAR study (illustrated by empty symbols), two different dependences in relation to the position and the type of substituents can be observed. Based on Figure 4A, it can be stated that compounds substituted in positions C_(3)_’, C_(3,4)_’, C_(3,5)_’, or C_(2,6)_’ showed an increasing trend of activity with the lipophilicity increase up to compound **6** (R = 3-CF_3_), at which the activity achieved plateau and from approximately log *k* ≈ 0.5 had an insignificant increase. The second, in fact, a linear, insignificantly increasing dependence can be found for the compounds substituted in positions C_(2)_’ and C_(2,5)_’. It is important to note that the antitubercular activity of the discussed cinnamanilides is also dependent on electronic σ parameters, see Figure 4B. As mentioned above, the linear insignificantly increasing dependence can be found for the derivatives substituted on the anilide in positions C_(2)_’ and C_(2,5)_’, while a bilinear dependence of activity on σ (for derivatives substituted in positions C_(3)_’, C_(3,4)_’, C_(3,5)_’, and C_(2,6)_’) can be observed. The activity increases with the increasing electron-withdrawing effect with *r* = 0.8803 (*n* = 5) to optimum σ_Ar_ ca. 1 (compound **13**, R = 3,5-CF_3_) and then decreases (*r* = 0.9162, *n* = 4) with increasing values of the electron-withdrawing parameter.

Additionally, a standard MTT (3-(4,5-dimethylthiazol-2-yl)-2,5-diphenyltetrazolium bromide) assay was performed on selected compounds that were the most effective against *M. tuberculosis* H37Ra and the MICs of which were previously determined, see Table 2. The MTT test can be used to assess cell growth by measuring respiration. The MTT measured viability of *M. tuberculosis* H37Ra less than 70% after exposure to the MIC values for each test agent is considered as a positive result of this assay. This low level of cell viability indicates inhibition of cell growth by inhibition of respiration [59]. All the selected compounds, i.e., **6** (R = 3-CF_3_, 40.99%), **10** (R = 3,4-Cl, 59.65%), **11** (R = 3,5-Cl, 22.65%), and **13** (R = 3,5-CF_3_, 66.09%) showed less than 70% viability of *M. tuberculosis* H37Ra at the tested concentration equal to MICs (i.e., 8 µg/mL or 22 and 27 µM). At MIC = 16 µg/mL (33 µM) compound **2** (R = 3-CH_3_) showed inhibition of viability 13.23%, and compound **5** (R = 3-F) showed inhibition of viability 11.04% at MIC = 32 µg/mL (33 µM).

Similar effects were observed previously, for example, with ring-substituted 6-hydroxynaphthalene-2-carboxanilides, where 3-Cl, 4-Cl, 3-Br, and 3-CF_3_ substituted derivatives decreased the viability of *M. tuberculosis* H37Ra in the range from 41.2% to 46.5% at the lowest tested concentration (MICs = 8 µg/mL) [20], with 8-hydroxy-*N*-(3-trifluoromethylphenyl)quinoline-2-carbox-amide, where the decrease of the viability of *M. tuberculosis* H37Ra was to 18.8% at MIC = 8 µg/mL [60], and with *N*-alkoxyphenylhydroxynaphthalenecarboxanilides substituted in C_(3)_’ position of the anilide core by a longer alkoxy tail [61,62]. Since the MTT assay was positive, it can be stated that the tested compounds caused a decrease of mycobacterial cell metabolism. Thus, based on the structure analogy of (2*E*)-*N*-aryl-3-phenylprop-2-enamides with naphthalene-2-carboxanilides, it may be hypothesized that the mechanism of action of these ring-substituted anilides of cinnamic acid could be connected with the affection of mycobacterial energy metabolism [59,63,64,65,66]; nevertheless, another possible site of action of the studied compounds in the mycobacteria cannot be excluded [67,68,69,70].

### 2.4. In Vitro Activity against Plant Pathogenic Fungi

Fungal infections are not only a problem in human and veterinary medicine, but also an important problem in agriculture. Plants diseases in general are a major factor limiting the crop quality. Fungal pathogens cause production losses and also can product mycotoxins, which are dangerous for consumers. The widespread use of fungicides increases food availability and safety, but it can leads to the selection of resistant pathogens and an increase of the production of mycotoxins [71]. As cinnamic acid and its derivatives do not have only antibacterial and antimycobacterial activity, but also activity against plant pathogens [7,72], all the prepared compounds were tested for their potency against *Fusarium avenaceum* (Fr.) Sacc. IMI 319947 and *Bipolaris sorokiniana* (Sacc.) Shoemaker H-299. *B. sorokiniana* is a wide-spread wheat and barley pathogen. It causes many diseases, such as head blight, seedling blight, common root rot, spot blotch, etc. [73]. The last one is a big problem, especially in Southern Asia, where 20% of crop yield is lost because of leaf blight disease [74]. *F. avenaceum* is one of the most common *Fusarium* species causing head blight disease of cereals. It can be isolated from cereal seeds and feed products [75]. *Fusarium* spp. produces a wide spectrum of mycotoxins. The most important are the trichothecenes, zearalenone, moniifromin, and the fumonisins. These compounds have toxic effect on humans and animals [76].

Only compound **6** (R = 3-CF_3_) showed moderate activity (MIC = 54.93 µM) against *F. avenaceum* within the series of compounds, see Table 2. On the other hand, the investigated compounds demonstrated higher effect against *B. sorokiniana*. (2*E*)-*N*-(3-Fluorophenyl)- (**5**) and (2*E*)-*N*-(3-methylphenyl)-3-phenylprop-2-enamide (**2**) had MICs = 16.58 and 33.71 µM, respectively, which is comparable with the benomyl standard. Also compounds **6**, **4** (R = 2-F), and **14** (2-F-5-Br) demonstrated moderate activity (MIC range 49.98–66.32 µM) against *B. sorokiniana.* Surprising was the inactivity of compound **13** (R = 3,5-CF_3_) against both fungal pathogens.

**Figure 5:**
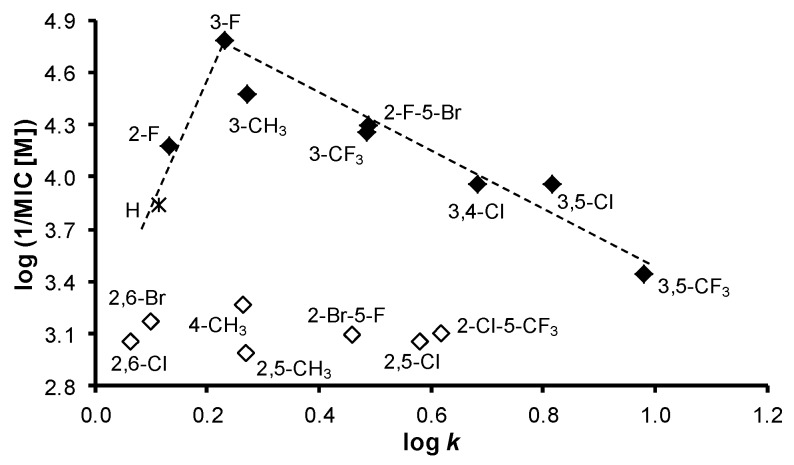
Relationships between in vitro antifungal activity against *B. sorokiniana* log (1/MIC (M)) and lipophilicity expressed as log *k* of studied compounds. (Derivatives excluded from SAR are illustrated by empty symbols.).

The dependences of the antifungal activity of the compounds against *B. sorokiniana* expressed as log (1/MIC (M)) on lipophilicity expressed as log *k* are illustrated in Figure 5. In general, effective compounds are preferentially substituted in positions C_(3)_’, C_(3,5)_’, or C_(3,4)_’. When inactive compounds (illustrated by empty symbols) substituted in C_(4)_’, C_(2,6)_’, or C_(2,5)_’ are eliminated from the SAR study, a bilinear dependence can be found. The activity increases with increasing lipophilicity from unsubstituted derivative **1** to compound **5** (R = 3-F) with the supposed lipophilicity optimum log *k* = 0.23 and then decreases to derivative **13** (R = 3,5-CF_3_); *r* = 0.9678, *n* = 7. It can be stated that it is an opposite trend in comparison with antitubercular findings, which can be caused by differences in the composition and structure of mycobacterial and fungal cell walls [77]. A similar trend can be found for electronic properties of substituents in individual derivatives. The activity increases with an increase of electron-withdrawing effect from compound **2** (R = 3-CH_3_, σ_Ar_ = 0.48) to an optimum σ_Ar_ = 0.82 (compound **5**, R = 3-F) and then decreases with increasing electron-withdrawing effect as follows: σ_Ar_ = 0.89 (compound **6**, R = 3-CF_3_), 1.02 (compound **4**, R = 2-F), 1.11 (compound **11**, R = 3,5-Cl), and 1.19 (compound **10**, R = 3,4-Cl).

#### Inhibition of *B. sorokiniana* Germination

All the compounds were additionally evaluated for the inhibition of *B. sorokiniana* conidium germination at two concentrations (128 and 256 μg/mL), see results in Table 4. It can be stated that at both concentrations, the compounds showed the inhibition of germination. The effect was concentration-dependent for compounds **7**, **10**, **15**, and **16**, and the rest of compounds had concentration-independent effect on the germination. Interestingly, compound **6** that was one of the most active against all bacterial cells including *M. tuberculosis* and displayed strong inhibition on the mycelial growth of both investigated fungi (*F. avenaceum* and *B. sorokiniana)* (Table 2), showed the weakest effect in the germination test. Apart from compound **6**, compounds **2**, **4**, **5**, and **14** exerted the highest *B. sorokiniana* mycelial growth inhibitory effect and had characteristic anti-germination activity. Moreover, compound **5** was the most effective in both assays (IC_50_ = 16.58 µM in mycelial growth test and 95.4% germination inhibition at 128 µg/mL).

### 2.5. In Vitro Antiproliferative Assay

The preliminary in vitro screening of the antiproliferative activity of the most effective antimicrobial compounds was performed using a Water Soluble Tetrazolium salts-1 (WST-1) assay kit [78] and the human monocytic leukemia THP-1 cell line by means of the method described recently [20,79]. The principle of the WST-1 assay kit is that antiproliferative compounds inhibit mitochondrial dehydrogenases. The activity of this enzyme directly correlates with the number of metabolically active cells in the culture. Antiproliferative effect was evaluated as IC_50_ value (concentration of compound causing 50% inhibition of cell proliferation). It can be stated that a compound is considered cytotoxic if it shows a toxic effect on cells up to 10 μM [80]. The highest compound concentration used for the toxicity test was 3-fold higher than this.

IC_50_ values of the most effective compounds **2** (R = 3-CH_3_), **5** (R = 3-F), **6** (R = 3-CF_3_), **10** (R = 3,4-Cl), **11** (R = 3,5-Cl), and **13** (R = 3,5-CF_3_) ranged from ca. 22 to >30 µM, see Table 2. For comparison, the IC_50_ of camptothecin was 0.16 ± 0.07 µM. Both compounds **5** and **2** effective against *B. sorokiniana* as well as compounds **10** and **11** potent against *M. tuberculosis* showed IC_50_ approximately 30 µM and higher, and compounds **6** and **13** showed IC_50_ = 22 µM, i.e., the treatment with these concentrations did not lead to significant antiproliferative effect on THP-1 cells, and these compounds inhibited selectively vital processes in *B. sorokiniana*, *M. tuberculosis*, or *Staphylococcus* strains. Based on these observations, it can be concluded that all the tested compounds can be considered as nontoxic agents for subsequent design of novel therapeutic agents.

### 2.6. Inhibition of Photosynthetic Electron Transport (PET) in Spinach Chloroplasts

The activity of the evaluated cinnamamides related to the inhibition of photosynthetic electron transport (PET) in spinach (*Spinacia oleracea* L.) chloroplasts was moderate or low relative to the standard, see Table 2, except for compound **11** (R = 3,5-Cl) that expressed the highest PET-inhibiting activity comparable with the Diuron^®^ standard (IC_50_ = 5.1 µM). With respect to these low activities, no detailed SAR study can be proposed; nevertheless, from the results listed in Table 2, bilinear trends for both PET inhibition vs. lipophilicity and PET inhibition vs. electronic properties of the anilide core can be suggested. Thus, PET inhibition activity increases with the lipophilicity (log *k*) increase as follows: 0.063 (**9**, R = 2,6-Cl) < 0.264 (**3**, R = 4-CH_3_) < 0.487 (**14**, R = 2-F-5-Br) < 0.682 (**10**, R = 3,4-Cl) <<< 0.815 (**11**, R = 3,5-Cl) and then decreases to 0.981 (**13**, R = 3,5-CF_3_). Also PET inhibition activity increases with the increase of electron-withdrawing (σ_Ar_) properties as follows: 0.46 (**3**, R = 4-CH_3_) < 1.05 (**13**, 3,5-CF_3_) <<< 1.11 (**11**, R = 3,5-Cl) and decreases as follows: >>> 1.19 (**10**, R = 3,4-Cl) > 1.28 (**14**, R = 2-F-5-Br) >> 1.33 (**9**, R = 2,6-Cl). It can be concluded, as mentioned above, that the substitution of the anilide core in C_(3,5)_’ or C_(3,4)_’ positions is preferable for high PET-inhibiting activity.

The inhibition of electron transport in PS II at the Q_B_ site plastoquinone, i.e., at the acceptor side of PS II was observed for ring-substituted salicylanilides and carbamoylphenylcarbamates [26,28], ring-substituted hydroxynaphthalene-2-carboxanilides [27,30], *N*-alkoxyphenylhydroxynaphthalene-carboxamides [29], 8-hydroxyquinoline-2-carboxamides [81], and *N*-substituted 2-aminobenzothiazoles [82]. Based on the structural analogy and the presence of the amide bond, it can be hypothesized that the mechanism of action of the investigated compounds is not different from the mechanism of action of the above compounds. Moreover, as mentioned previously [26,27,28,29,83,87], a good correlation between antimycobacterial activity and herbicidal effect was found.

### 2.7. In Vivo Toxicity against Plant Cells

**Figure 6:**
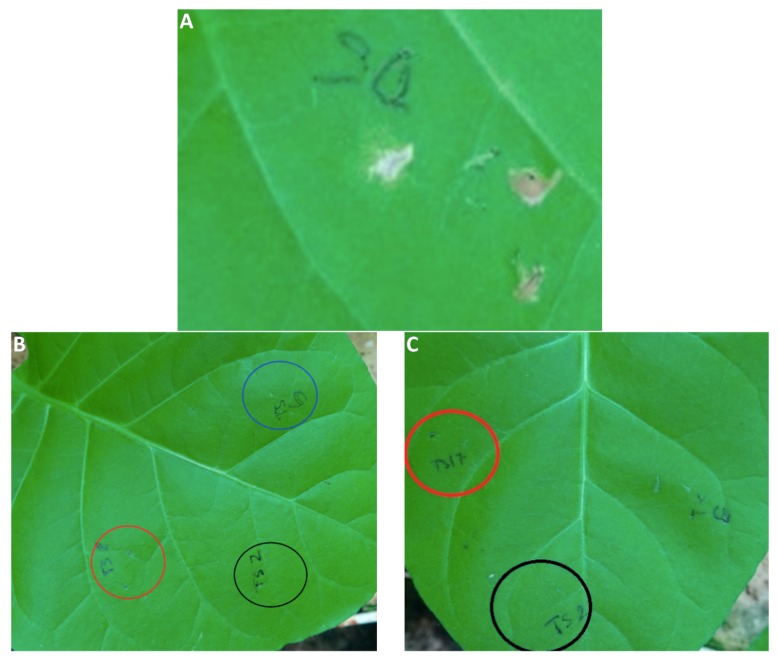
Toxic effect of 5% DMSO (**A**) to leaf of *Nicotiana tabacum* compared to nontoxic effect of compounds **2** (blue ring), **5** (red ring), and **6** (black ring) (**B**) and nontoxic effect of compounds **6** (black ring) and **14** (red ring) (**C**). The black spots in the injected area of compound **5** in Figure 6B are damages of the leaf tissue caused by a needle.

The most potent antifungal compounds **2**, **5**, **6**, and **14** were tested for in vivo toxicity against plant cells. *Nicotiana tabacum* var. Samsun was used for this test [88]. The results of treatment of plant leaves with injected water solutions of each compound as well as dimethyl sulfoxide (DMSO) are illustrated in Figure 6, where photographs of leaves are shown. The negative DMSO control did not have any toxic effect to the plant cells as well. On the other hand, the 5% aqueous solution of DMSO showed a significant toxic effect on the leaves demonstrated as a loss of chlorophyll in the injected area (Figure 6A). Based on Figure 6B,C, it can be concluded that the most effective antifungal compounds had no visible influence on the plant tissue.

These results correspond to above-mentioned PET-inhibiting activity, when all the tested compounds showed no PET inhibition, and thus, they will not be toxic for plants at their prospective application as a plant fungicide.

## 3. Materials and Methods

### 3.1. Chemistry—General Information

All reagents were purchased from Merck (Sigma-Aldrich, St. Louis, MO, USA) and Alfa (Alfa-Aesar, Ward Hill, MA, USA). Reactions were performed using a CEM Discover SP microwave reactor (CEM, Matthews, NC, USA). Melting points were determined on an apparatus Stuart SMP10 (Stone, UK) and are uncorrected. Infrared (IR) spectra were recorded on an UATR Zn/Se for a Spectrum Two™ Fourier-transform IR spectrometer (PerkinElmer, Waltham, MA, USA). The spectra were obtained by the accumulation of 32 scans with 4 cm^−1^ resolution in the region of 4000–400 cm^−1^. All ^1^H- and ^13^C-NMR spectra were recorded on a JEOL ECZR 400 MHz NMR spectrometer (400 MHz for ^1^H and 100 MHz for ^13^C, Jeol, Tokyo, Japan) in dimethyl sulfoxide-*d_6_* (DMSO-*d_6_*). ^1^H and ^13^C chemical shifts (δ) are reported in ppm. High-resolution mass spectra were measured using a high-performance liquid chromatograph Dionex UltiMate^®^ 3000 (Thermo Scientific, West Palm Beach, FL, USA) coupled with an LTQ Orbitrap XL^TM^ Hybrid Ion Trap-Orbitrap Fourier Transform Mass Spectrometer (Thermo Scientific) equipped with a HESI II (heated electrospray ionization) source in the positive mode.

#### Synthesis

Cinnamic acid (3.37 mM) was suspended at room temperature in dry chlorobenzene (20 mL) inside a microwave tube, where phosphorus trichloride (1.7 mM) and the corresponding aniline (3.37 mM) were added dropwise. Then a magnetic stirrer was used, and the reaction mixture was transferred to the microwave reactor at 120 °C for 20 min, where the synthesis at elevated pressure was performed. After the mixture was cooled to 60 °C, and solvent was evaporated in vacuum. A solid was washed with 2 M HCl, and a crude product was recrystallized, first using 96% ethanol and then using 50% ethanol.

*(2E)-N-Phenyl-3-phenylprop-2-enamide* (**1**) [89]. Yield 91%; Mp 116–118 °C; IR (cm^−1^): 3062, 3028, 1661, 1626, 1594, 1578, 1543, 1493, 1442, 1347, 1288, 1248, 1188, 976, 904, 864, 759, 737, 704, 690, 676, 620, 592, 553, 512, 484, 454; ^1^H-NMR (DMSO-*d_6_*), δ: 10.22 (s, 1H), 7.72 (d, *J* = 7.3 Hz, 2H), 7.64–7.59 (m, 3H), 7.47–7.36 (m, 3H), 7.34 (t, *J* = 8 Hz, 2H), 7.09–7.05 (m, 1H), 6.85 (d, *J* = 16 Hz, 1H); ^13^C-NMR (DMSO-*d*_6_), δ: 163.53, 140.16, 139.28, 134.73, 129.77, 129.03, 128.81, 127.72, 123.36, 122.29, 119.23; HR-MS: for C_15_H_14_NO [M + H]^+^ calculated 224.1070 *m*/*z*, found 224.1065 *m*/*z*.

*(2E)-N-(3-Methylphenyl)-3-phenylprop-2-enamide* (**2**) [89]. Yield 80%; Mp 113–115 °C; IR (cm^−1^): 3259, 3136, 3082, 3064, 3028, 2918, 1658, 1620, 1541, 1488, 1447, 1408, 1342, 1292, 1201, 987, 978, 864, 775, 763, 729, 714, 685, 676, 556, 487; ^1^H-NMR (DMSO-*d*_6_), δ: 10.14 (s, 1H), 7.64–7.60 (m, 2H), 7.58 (d, *J* = 15.6 Hz, 1H), 7.53 (m, 1H), 7.5 (d, *J* = 8.2 Hz, 1H), 7.47–7.40 (m, 3H), 7.21 (t, *J* = 7.8 Hz, 1H), 6.89 (d, *J*=7.8 Hz, 1H), 6.84 (d, *J* = 15.6 Hz, 1H), 2.3 (s, 3H); ^13^C-NMR (DMSO-*d_6_*), δ: 163.48, 140.06, 139.23, 137.99, 134.75, 129.78, 129.04, 128.67, 127.73, 124.11, 122.38, 119.73, 116.45, 21.25; HR-MS: for C_16_H_16_NO [M + H]^+^ calculated 238.1226 *m*/*z*, found 238.1222 *m*/*z*.

*(2E)-N-(4-Methylphenyl)-3-phenylprop-2-enamide* (**3**) [89]. Yield 82%; Mp 166–168 °C; IR (cm^−1^): 3240, 3187, 3128, 3085, 3028, 2954, 1660, 1622, 1597, 1538, 1493, 1448, 1405, 1342, 1252, 1187, 984, 973, 813, 781, 762, 720, 674, 533, 510, 485; ^1^H-NMR (DMSO-*d*_6_) δ: 10.14 (s, 1H), 7.63–7.55 (m, 5H), 7.47–7.40 (m, 3H), 7.15–7.13 (m, 2H), 6.83 (d, *J* = 16 Hz, 1H), 2.26 (s, 3H); ^13^C-NMR (DMSO-*d*_6_) δ: 163.33, 139.90, 136.81, 134.79, 132.32, 129.73, 129.22, 129.03, 127.70, 122.40, 119.22, 20.51; HR-MS: for C_16_H_16_NO [M + H]^+^ calculated 238.1226 *m/z*, found 238.1222 *m/*z.

*(2E)-N-(2-Fluorophenyl)-3-phenylprop-2-enamide* (**4**) [89]. Yield 85%; Mp 114–116 °C; IR (cm^−1^): 3238, 3182, 3126, 3085, 3059, 3020, 1661, 1627, 1539, 1491, 1448, 1342, 1257, 1188, 1105, 973, 760, 731, 719, 701, 687, 659, 557, 535, 495, 479; ^1^H-NMR (DMSO-*d*_6_) δ: 9.69 (s, 1H), 8.11 (td, *J* = 7.9 Hz, 2.1 Hz, 1H), 7.64–7.57 (m, 3H), 7.48–7.40 (m, 4H), 7.31–7.26 (m, 1H), 7.20–7.16 (m, 1H), 7.09 (d, *J*=15.6 Hz, 1H); ^13^C-NMR (DMSO-*d*_6_) δ: 163.96, 153.34 (d, *J* = 244.7 Hz), 140.72, 134.74, 129.90, 129.05, 127.82, 126.41 (d, *J* = 10.6 Hz), 125.09 (d, *J* = 7.7 Hz), 124.44 (d, *J* = 2.9 Hz), 121.88, 119.23, 115.47 (d, *J* = 19.3 Hz); HR-MS: for C_15_H_13_FNO [M + H]^+^ calculated 242.0976 *m/z*, found 242.0971 *m/z*.

*(2E)-N-(3-Fluorophenyl)-3-phenylprop-2-enamide* (**5**). Yield 84%; Mp 112–114 °C; IR (cm^−1^): 3298, 3061, 3031, 2979, 1625, 1596, 1529, 1489, 1420, 1334, 1186, 974, 943, 858, 777, 768, 756, 727, 714, 688, 679, 658, 556, 492; ^1^H-NMR (DMSO-*d*_6_) δ: 10.44 (s, 1H), 7.76–7.72 (m, 1H), 7.65–7.61 (m, 3H), 7.48–7.34 (m, 5H), 6.92–6.88 (m, 1H), 6.82 (d, *J* = 15.6 Hz, 1H); ^13^C-NMR (DMSO-*d*_6_) δ: 163.87, 162.23 (d, *J* = 241.8 Hz), 141.00 (d, *J* = 10.6 Hz), 140.80, 134.61, 130.44 (d, *J* = 9.6 Hz), 129.95, 129.06, 127.85, 121.87, 115.02 (d, J=2.9 Hz), 109.83 (d, *J* = 21.2 Hz), 106.07 (d, *J* = 27.3 Hz); HR-MS: for C_15_H_13_FNO [M + H]^+^ calculated 242.0976 *m/z*, found 242.0972 *m/z*.

*(2E)-3-Phenyl-N-[3-(trifluoromethyl)phenyl]prop-2-enamide* (**6**) [90]. Yield 75%; Mp 109–111 °C; IR (cm^−1^): 3400, 2905, 2360, 1678, 1630, 1526, 1491, 1331, 1154, 1112, 1100, 1072, 982, 886, 808; ^1^H-NMR (DMSO-*d*_6_) δ: 10.57 (s, 1H), 8.22 (s, 1H), 7.87 (d, *J* = 8.2 Hz, 1H), 7.66–7.62 (m, 3H), 7,58 (t, *J* = 7.8 Hz, 1H), 7.48–7.40 (m, 4H), 6.82 (d, *J* = 15.6 Hz, 1H); ^13^C-NMR (DMSO-*d*_6_) δ: 164.05, 140.98, 140.07, 134.54, 130.04, 129.99, 129.58 (q, *J* = 30.8 Hz), 129.05, 127.88, 124.16 (q, *J* = 272.6 Hz), 122.76, 121.72, 119.65 (q, *J* = 3.9 Hz), 115.29 (q, *J* = 3.9 Hz); HR-MS: for C_16_H_13_F_3_NO [M + H]^+^ calculated 292.0944 *m/z*, found 292.0938 *m/z*.

*(2E)-N-(2,5-Dimethylphenyl)-3-phenylprop-2-enamide* (**7**). Yield 91%; Mp 174–176 °C; IR (cm^−1^): 3242, 3056, 3028, 2977, 2918, 1657, 1621, 1579, 1544, 1494, 1449, 1417, 1343, 1286, 1266, 1199, 1159, 990, 978, 878, 780, 764, 746, 734, 724, 707, 681, 624, 560, 492; ^1^H-NMR (DMSO-*d*_6_) δ: 9.42 (s, 1H), 7.63 (d, *J* = 6.9 Hz, 2H), 7.58 (d, *J* = 16 Hz, 1H), 7.47–7.39 (m, 4H), 7.10 (d, *J* = 7.8 Hz, 1H), 6.99 (d, *J* = 16 Hz, 1H), 6.90 (d, *J* = 7.3 Hz, 1H), 2.26 (s, 3H), 2.20 (s, 3H); ^13^C-NMR (DMSO-*d*_6_) δ: 163.58, 139.88, 136.17, 134.97, 134.85, 130.15, 129.67, 128.99, 127.94, 127.68, 125.70, 124.97, 122.36, 20.67, 17.54; HR-MS: for C_17_H_18_NO [M + H]^+^ calculated 252.1383 *m/z*, found 252.1378 *m/z*.

*(2E)-N-(2,5-Dichlorophenyl)-3-phenylprop-2-enamide* (**8**). Yield 83%; Mp 173–175 °C; IR (cm^−1^): 3234, 3108, 3062, 3026, 1662, 1622, 1582, 1528, 1463, 1449, 1406, 1341, 1261, 1182, 1093, 1055, 992, 979, 917, 872, 856, 806, 761, 718, 693, 675, 580, 559, 488; ^1^H-NMR (DMSO-*d*_6_) δ: 9.77 (s, 1H), 8.15 (d, *J* = 2.3 Hz, 1H), 7.66–7.62 (m, 3H), 7.54 (d, *J* = 8.7 Hz, 1H), 7.45–7.41 (m, 3H), 7.24 (dd, *J* = 8.7 Hz, 2.7 Hz, 1H), 7.18 (d, *J* = 15.6 Hz, 1H); ^13^C-NMR (DMSO-*d*_6_) δ: 164.20, 141.47, 136.28, 134.60, 131.61, 130.83, 130.05, 129.03, 127.94, 125.40, 124.11, 123.61, 121.51; HR-MS: for C_18_H_12_Cl_2_NO [M + H]^+^ calculated 292.0290 *m/z*, found 292.0289 *m/z*.

*(2E)-N-(2,6-Dichlorophenyl)-3-phenylprop-2-enamide* (**9**). Yield 85%; Mp 212–214 °C; IR (cm^−1^): 3256, 1661, 1630, 1569, 1522, 1449, 1428, 1338, 1183, 971, 782, 758, 712, 697, 508; ^1^H-NMR (DMSO-*d*_6_) δ: 10.09 (s, 1H), 7.67–7.62 (m, 3H), 7.58–7.56 (m, 2H), 7.48–7.41 (m, 3H), 7.39–7.35 (m, 1H), 6.89 (d, *J* = 16 Hz, 1H); ^13^C-NMR (DMSO-*d*_6_) δ: 163.67, 140.98, 134.51, 133.69, 133.03, 129.97, 129.19, 129.05, 128.56, 127.85, 120.72; HR-MS: for C_15_H_12_Cl_2_NO [M + H]^+^ calculated 292.0290 *m/z*, found 292.0288 *m/z*.

*(2E)-N-(3,4-Dichlorophenyl)-3-phenylprop-2-enamide* (**10**). Yield 78%; Mp 173–175 °C; IR (cm^−1^): 3269, 3095, 3025, 1663, 1627, 1585, 1526, 1474, 1378, 1339, 1289, 1229, 1182, 1126, 1025, 974, 863, 810, 761, 699, 677, 579, 564, 515, 484; ^1^H-NMR (DMSO-*d*_6_) δ: 10.52 (s, 1H), 8.12–8.11 (m, 1H), 7.64–7.61 (m, 3H), 7.58 (s, 2H), 7.46–7.40 (m, 3H), 6.79 (d, *J* = 16 Hz, 1H); ^13^C-NMR (DMSO-*d*_6_) δ: 163.63, 141.08, 139.38, 134.49, 131.09, 130.72, 130.03, 129.05, 127.88, 124.78, 121.55, 120.39, 119.25; HR-MS: for C_15_H_12_Cl_2_NO [M + H]^+^ calculated 292.0290 *m/z*, found 292.0288 *m/z*.

*(2E)-N-(3,5-Dichlorophenyl)-3-phenylprop-2-enamide* (**11**) [89]. Yield 79%; Mp 139–141 °C; IR (cm^−1^): 3246, 3177, 3085, 2967, 1663, 1622, 1584, 1532, 1442, 1408, 1338, 1180, 1109, 969, 937, 844, 802, 760,717, 668, 556, 484; ^1^H-NMR (DMSO-*d*_6_) δ: 10.56 (s, 1H), 7.76 (d, *J* = 1.8 Hz, 2H), 7.65–7.61 (m, 3H), 7.48–7.40 (m, 3H), 7.29 (t, *J* = 2,1 Hz, 1H), 6.76 (d, J = 15.6 Hz, 1H); ^13^C-NMR (DMSO-*d*_6_) δ: 164.06, 141.60, 141.35, 134.41, 134.12, 130.06, 129.02, 127.90, 122.48, 121.37, 117.30; HR-MS: for C_15_H_12_Cl_2_NO [M + H]^+^ calculated 292.0290 *m/z*, found 292.0288 *m/z*.

*(2E)-N-(2,6-Dibromophenyl)-3-phenylprop-2-enamide* (**12**). Yield 81%; Mp 240–242 °C; IR (cm^−1^): 3251, 3180, 3028, 2902, 1660, 1627, 1558, 1516, 1441, 1422, 1337, 1263, 1180, 970, 781, 766, 759, 721, 710, 694, 686, 626, 561, 501; ^1^H-NMR (DMSO-*d*_6_) δ: 10.12 (s, 1H), 7.75 (d, *J* = 7.8 Hz, 2H), 7.67–7.64 (m, 2H), 7.60 (d, *J* = 16 Hz, 1H), 7.48–7.40 (m, 3H), 7.21 (t, *J* = 8 Hz, 1H), 6.87 (d, *J* = 15.6 Hz, 1H); ^13^C-NMR (DMSO-*d*_6_) δ: 163.50, 140.85, 135.77, 134.50, 132.24, 130.19, 129.94, 129.05, 127.80, 124.33, 120.86; HR-MS: for C_15_H_12_Br_2_NO [M + H]^+^ calculated 379.9280 *m/z*, found 379.9288 *m/z*.

*(2E)-N-[3,5-bis(Trifluoromethyl)phenyl]-3-phenylprop-2-enamide* (**13**). Yield 75%; Mp 143–145 °C; IR (cm^−1^): 3272, 3085, 2967, 2938, 2879, 1663, 1622, 1575, 1472, 1440, 1377, 1276, 1168, 1129, 1110, 1096, 974, 937, 886, 859, 841, 728, 680, 627, 557, 486; ^1^H-NMR (DMSO-*d*_6_) δ: 10.89 (s, 1H), 8.36 (s, 2H), 7.77 (s, 1H), 7.70–7.65 (m, 3H), 7.48–7.40 (m, 3H), 6.78 (d, *J* = 16 Hz, 1H); ^13^C-NMR (DMSO-*d*_6_) δ: 164.42, 141.78, 141.13, 134.28, 130.80 (q, *J* = 32.8 Hz), 130.22, 129.08, 127.98, 123.23 (q, *J* = 272.6 Hz), 121.07, 118.93–118.78 (m), 116.12–115.98 (m); HR-MS: for C_17_H_12_F_6_NO [M + H]^+^ calculated 360.0818 *m/z*, found 360.0811 *m/z*.

*(2E)-N-(2-Fluoro-5-bromophenyl)-3-phenylprop-2-enamide* (**14**). Yield 83%; Mp 161–164 °C; IR (cm^−1^): 3280, 2967, 2936, 2879, 1660, 1613, 1529, 1474, 1448, 1411, 1345, 1254, 1174, 985, 870, 800, 762, 722, 676, 617, 600, 564, 484; ^1^H-NMR (DMSO-*d*_6_) δ: 10.13 (s, 1H), 8.44 (dd, *J* = 7.1 Hz, 2.5 Hz, 1H), 7.65–7.60 (m, 3H), 7.48–7.40 (m, 3H), 7.35–7.27 (m, 2H), 7.11 (d, *J* = 15.6 Hz, 1H); ^13^C-NMR (DMSO-*d*_6_) δ: 164.22, 152.03 (d, *J* = 245.6 Hz), 141.30, 134.60, 130.02, 129.04, 128.25 (d, *J* = 12.5 Hz), 127.88, 127.10 (d, *J* = 7.7 Hz), 125.10, 121.50, 117.41 (d, *J* = 21.2 Hz), 115.91 (d, *J* = 2.9 Hz); HR-MS: for C_15_H_12_FBrNO [M + H]^+^ calculated 320.0081 *m/z*, found 320.0077 *m/z*.

*(2E)-N-(2-Bromo-5-fluorophenyl)-3-phenylprop-2-enamide* (**15**). Yield 81%; Mp 164–166 °C; IR (cm^−1^): 3230, 3072, 3045, 2967, 2937, 2880, 1661, 1623, 1591, 1538, 1420, 1342, 1198, 1155, 991, 977, 869, 854, 804, 761, 715, 668, 598, 589, 559, 482; ^1^H-NMR (DMSO-*d*_6_) δ: 9.64 (s, 1H), 7.83 (dd, *J* = 11 Hz, 3.2 Hz, 1H), 7.72 (dd, *J* = 8.7 Hz, 5.9 Hz, 1H), 7.67–7.62 (m, 3H), 7.48–7.42 (m, 3H), 7.15 (d, *J* = 15.6 Hz, 1H), 7.04 (ddd, *J* = 8.9 Hz, 8 Hz, 3.2 Hz, 1H); ^13^C-NMR (DMSO-*d*_6_) δ: 164.12, 161.05 (d, *J* = 243.7 Hz), 141.44, 137.74 (d, *J* = 11.6 Hz), 134.61, 133.85 (d, *J* = 8.7 Hz), 130.05, 129.04, 127.95, 121.55, 113.41 (d, *J* = 23.1 Hz), 112.44 (d, *J* = 27 Hz), 110.6 (d, *J* = 2.9 Hz); HR-MS: for C_15_H_12_FBrNO [M + H]^+^ calculated 320.0081 *m/z*, found 320.0077 *m/z*.

*(2E)-N-[2-Chloro-5-(trifluoromethyl)phenyl]-3-phenylprop-2-enamide* (**16**). Yield 77%; Mp 144–146 °C; IR (cm^−1^): 3280, 2967, 2936, 2879, 1528, 1329, 1262, 1165, 1116, 1080, 964, 892, 814, 758, 707, 686, 668, 644, 605, 560, 534, 490, 452; ^1^H-NMR (DMSO-*d*_6_) δ: 9,22 (s, 1H), 8.40 (d, *J* = 1.8 Hz, 1H), 7.79–7.77 (m, 1H), 7.68–7.64 (m, 3H), 7.55–7.53 (m, 1H), 7.49–7.40 (m, 3H), 7.19 (d, *J* = 15.6 Hz, 1H); ^13^C-NMR (DMSO-*d*_6_) δ: 164.42, 141.66, 135.99, 134.60, 130.70, 130.06, 129.02, 128.98, 128.09 (q, *J* = 38.2 Hz), 127.96, 123.69 (q, *J* = 272.6 Hz), 121.98 (q, *J* = 3.9 Hz), 121.46, 120.95 (q, *J* = 3.9 Hz); HR-MS: for C_16_H_12_ClF_3_NO [M + H]^+^ calculated 326.0554 *m/z*, found 326.0547 *m/z*.

### 3.2. Lipophilicity Determination by HPLC (Capacity Factor k/Calculated log k)

A HPLC separation module Waters^®^ e2695 equipped with a Waters 2996 PDA Detector (Waters Corp., Milford, MA, USA) were used. A chromatographic column Symmetry^®^ C_18_ 5 μm, 4.6 × 250 mm, Part No. W21751W016 (Waters Corp.) was used. The HPLC separation process was monitored by the Empower^™^ 3 Chromatography Data Software (Waters Corp.). Isocratic elution by a mixture of MeOH p.a. (72%) and H_2_O-HPLC Mili-Q grade (28%) as a mobile phase was used. The total flow of the column was 1.0 mL/min, injection 5 μL, column temperature 40 °C, and sample temperature 10 °C. The detection wavelength 214 nm was chosen. The KI methanolic solution was used for the determination of dead time (t_D_). Retention times (*t_R_*) were measured in minutes. The capacity factors *k* were calculated using the Empower^™^ 3 Chromatography Data Software according to the formula *k* = (*t_R_* − *t_D_*)/*t_D_*, where *t_R_* is the retention time of the solute, while *t_D_* is the dead time obtained using an unretained analyte. Each experiment was repeated three times. Log *k*, calculated from the capacity factor *k*, is used as the lipophilicity index converted to log *P* scale [36]. The log *k* values of individual compounds are shown in Table 1.

### 3.3. Biological Testing

#### 3.3.1. In Vitro Antibacterial Evaluation

The synthesized compounds were evaluated for in vitro antibacterial activity against representatives of multidrug-resistant bacteria and clinical isolates of methicillin-resistant *Staphylococcus aureus* (MRSA) 63718, SA 630, and SA 3202 [37,38] that were obtained from the National Institute of Public Health (Prague, Czech Republic). *S. aureus* ATCC 29213 was used as a reference and quality control strain. In addition, all the compounds were tested for their activity against vancomycin-susceptible *Enterococcus faecalis* ATCC 29212 as a reference strain and three isolates from American crows of vanA-carrying vancomycin-resistant *E. faecalis* (VRE) 342B, 368, and 725B [39]. Ampicillin (Sigma) was used as the standard. Prior to testing, each strain was passaged onto nutrient agar (Oxoid, Basingstoke, UK) with 5% of bovine blood, and bacterial inocula were prepared by suspending a small portion of bacterial colony in sterile phosphate buffered saline (pH 7.2–7.3). The cell density was adjusted to 0.5 McFarland units using a densitometer (Densi-La-Meter, LIAP, Riga, Latvia). This inoculum was diluted to reach the final concentration of bacterial cells 5 × 10^5^ CFU/mL in the wells. The compounds were dissolved in DMSO (Sigma), and the final concentration of DMSO in the Cation Adjusted Mueller-Hinton (CaMH) broth (Oxoid) or Brain-Heart Infusion for enterococci did not exceed 2.5% of the total solution composition. The final concentrations of the evaluated compounds ranged from 256 to 0.008 μg/mL. The broth dilution micro-method, modified according to the NCCLS (National Committee for Clinical Laboratory Standards) guidelines [91] in Mueller–Hinton (MH) broth, was used to determine the minimum inhibitory concentration (MIC). Drug-free controls, sterility controls, and controls consisting of MH broth and DMSO alone were included. The determination of results was performed visually after 24 h of static incubation in the darkness at 37 °C in an aerobic atmosphere. The results are shown in Table 2.

#### 3.3.2. Synergy Effect with Clinically Used Drugs

For synergy effect study, a method of fractional inhibitory concentration was used. The tested compounds (A) and conventional used antibiotic (B) (tetracycline, ciprofloxacin, and vancomycin (purchased from Sigma)) were diluted in the microtitration plate in CaMH broth (Oxoid) to get an original combination of concentration in every well. The raw H was used for evaluation of MIC_(A)_; column 12 was used for evaluation of MIC_(B)_. The plate was inoculated by the bacterial suspension to reach final concentration 5 × 10^5^ CFU/mL in the wells. The fractional inhibitory concentration (FIC) index was calculated using the concentrations in the first nonturbid (clear) well found in each row and column along the turbidity/nonturbidity interface [42]. A ΣFIC ≤ 0.5 means synergy; 0.5 < ΣFIC < 1 is additivity; 1 ≤ ΣFIC < 4 is indifference; and ΣFIC ≥ 4 is antagonism [41]. The tests were made in duplicate, and the results were averaged. The results are summarized in Table 3.

#### 3.3.3. Dynamics of Antibacterial Effect

The most active antistaphylococcal compounds were studied for their dynamics of antibacterial effect. The method of time-kill curves were used [37,40]. Compound **6** was diluted in CaMH to reach the final concentration equal to 1× MIC, 2× MIC, and 4× MIC. For the compound **13**, only concentrations 1× MIC and 2× MIC were used, due to the higher MIC and dissolution problems in the concentration equal to 4× MIC. The tubes were inoculated with the culture of *S. aureus* ATTC 29213 diluted to 1 McFarland in the exponential phase of growth. The final concentration of bacteria was 7.5 × 10^6^ CFU/mL. Tubes were stored in an incubator at 37 °C without shaking. Immediately after 4, 6, 8, and 24 h of inoculation, 100 μL of sample was serially diluted 1:10 in phosphate buffered saline (PBS). From each dilution 2 × 20 µL were put onto MH agar plates. The plates were incubated at 37 °C for 24 h, and the colonies were counted. Bactericidal effect is defined as a −3log decrease of CFU/mL compared to the growth control in time 0. The test was made in duplicate on 2 separate occasions, and the results were averaged. The results are illustrated in Figure 2.

#### 3.3.4. Biofilm Inhibition Assay

The most active antistaphylococcal compounds were studied for their ability to inhibit biofilm formation. The compounds were diluted in a 96-well plate in tryptic soy broth (TSB) containing 2% of glucose to reach concentrations 256–2 µg/mL. *S. aureus* ATCC 29213 cultivated overnight on blood agar was used for preparing the inoculum. A few colonies were put in a tube with 5 mL of TSB + 2% glucose and cultivated to reach the exponential phase of growth. The inoculum was diluted to 1 McFarland and then 1:1000 in TSB + 2% glucose. The final concentration of bacteria in each well was 1 × 10^5^. As positive controls, ampicillin, vancomycin, ciprofloxacin, and tetracycline (Sigma) were used. As the compounds were dissolved in DMSO (up to 5%), the growth control included 5% of DMSO for verification that the applied DMSO concentration did not possess bacterial growth-inhibiting activity. The plate was cultivated for 48 h at 37 °C without shaking. After incubation, the content of the wells was removed and the wells were washed 3-fold by PBS. After drying, 125 µL of 0.1% crystal violet was put to every well. The plane was stained for 20 min at room temperature, the content was removed, and the plate was again washed by PBS 3-fold. The coloured biofilm was taken off from the wells by 33% acetic acid, and absorbance in 595 nm was measured. As a blank, non-inoculated plate treated in the same way was used. The test was made in triplicates in 3 separated occasions. The ability to inhibit biofilm formation was evaluated as a percentage inhibition of growth compared to the growth control. The results are illustrated in Figure 3.

#### 3.3.5. In Vitro Antimycobacterial Evaluation

*Mycobacterium tuberculosis* ATCC 25177/H37Ra was grown in Middlebrook broth (MB), supplemented with Oleic-Albumin-Dextrose-Catalase (OADC) supplement (Difco, Lawrence, KS, USA). At log phase growth, a culture sample (10 mL) was centrifuged at 15,000 rpm/20 min using a bench top centrifuge (MPW-65R, MPW Med Instruments, Warszawa, Poland). Following the removal of the supernatant, the pellet was washed in fresh Middlebrook 7H9GC broth and resuspended in fresh, ODAC-supplemented MB (10 mL). The turbidity was adjusted to match McFarland standard No. 1 (3 × 10^8^ CFU) with MB broth. A further 1:10 dilution of the culture was then performed in MB broth. The antimicrobial susceptibility of *M. tuberculosis* was investigated in a 96-well plate format. In these experiments, sterile deionised water (300 µL) was added to all outer-perimeter wells of the plates to minimize evaporation of the medium in the test wells during incubation. Each evaluated compound (100 µL) was incubated with *M. tuberculosis* (100 µL). Dilutions of each compound were prepared in duplicate. For all synthesized compounds, final concentrations ranged from 128 to 4 μg/mL. All compounds were dissolved in DMSO, and subsequent dilutions were made in supplemented MB. The plates were sealed with Parafilm and incubated at 37 °C for 14 days. Following incubation, a 10% addition of alamarBlue (Difco) was mixed into each well, and readings at 570 nm and 600 nm were taken, initially for background subtraction and subsequently after 24 h reincubation. The background subtraction is necessary for strongly coloured compounds, where the colour may interfere with the interpretation of any colour change. For noninterfering compounds, a blue colour in the well was interpreted as the absence of growth, and a pink colour was scored as growth. Isoniazid (Sigma) was used as the positive control, as it is a clinically used antitubercular drug. The results are shown in Table 2.

#### 3.3.6. MTT Assay

Compounds were prepared as previously stated and diluted in Middlebrook media to achieve the desired final concentration 128–1 µg/mL. *Mycobacterium tuberculosis* ATCC 25177/H37Ra was suspended in ODAC supplemented Middlebrook broth at a MacFarland standard of 1.0 and then diluted 1:10, using Middlebrook broth as a diluent. The diluted mycobacteria (50 µL) were added to each well containing the compound to be tested. Diluted mycobacteria in broth free from inhibiting compounds were used as a growth control. As positive controls, ciprofloxacin and rifampicin were used. All compounds and controls were prepared in duplicate. Plates were incubated at 37 °C for 7 days. After the incubation period, 10% well volume of MTT (3-(4,5-dimethylthiazol-2-yl)-2,5-diphenyl-tetrazolium bromide) reagent (Sigma) was mixed into each well and incubated at 37 °C for 4 h in dark. Then 100 µL of 17% Sodium dodecyl sulfate in 40% dimethylformamide was added to each well. The plates were read at 570 nm. The absorbance readings from the cells grown in the presence of the tested compounds were compared with uninhibited cell growth to determine the relative percent viability. The percent viability was determined through the MTT assay. The percent viability is calculated through comparison of a measured value against that of the uninhibited control: % viability = OD_570E_/OD_570P_ × 100, where OD_570E_ is the reading from the compound-exposed cells, while OD_570P_ is the reading from the uninhibited cells (positive control). Cytotoxic potential is determined by a percent viability of <70% [59,92]. Rifampicin a ciprofloxacin (Sigma) were used as positive controls.

#### 3.3.7. In Vitro Antifungal Activity

96-Well microplates were used for testing the inhibitory effect of compounds against mycelial growth of *Fusarium avenaceum* (Fr.) Sacc. IMI 319947 and *Bipolaris sorokiniana* (Sacc.) Shoemaker H-299 (NCBI GenBank accession No. MH697869). The compounds were diluted in DMSO to reach the concentration of 10 mg/mL and then diluted in the microtiter plates in supplemented lysogeny broth (LB, 10 g/L tryptone (Microtrade, Budapest, Hungary), 5 g/L yeast extract (Scharlau, Barcelona, Spain), and 10 g/L NaCl (Reanal, Budapest, Hungary)) to get the final concentration of 256–2 µg/mL. Positive (benomyl, Chinoin Fundazol 50WP^®^, Chinoin, Budapest, Hungary) and negative (solvents of the compounds) controls were used. 50 mL LB medium was inoculated with the fungal culture grown in an agar plate and shaken at 100 rpm at 22 °C for 3 days in dark; then mycelium was cut with a sterile blender to small parts. The mycelium suspension was diluted to set OD_600_ = 0.2. This inoculum was diluted 2-fold with the content of the wells. The absorbance at 600 nm was measured by a spectrophotometer (Labsystems Multiscan MS 4.0, Thermo Scientific) immediately, and the plates were incubated at 22 °C. The absorbance was measured again after 24, 48, and 72 h. The MIC was counted in the time, when absorbance in the negative control tripled. The experiment was repeated on 3 separated occasions, and the results were averaged. The results are summarized in Table 2.

#### 3.3.8. Inhibition of *Bipolaris sorokiniana* Germination

The inhibitory effect on the conidium germination was tested according to De Lucca et al. [93] with some modifications. The tested compounds were diluted in a 96-well microplate in 45 µL sterile distilled water to reach the final concentrations of 256 and 128 µg/mL. As a positive control, benomyl (10 and 5 µg/mL) was used. As a negative control, nontreated wells with 5% DMSO were used. Conidium suspension was prepared from sporulating culture grown on an agar plate. 5 µL of the aliquot was added to each well. The final concentration of conidia in each well was 3000 conidia in 1 mL. The plate was incubated at 22 °C for 48 h in dark. After the incubation, the aliquot from each well was observed microscopically (400×) for germination. A total of 50 conidia from each well were observed. Germination was defined as the development of germ tube(s) of any size from a conidium. The inhibition effect was counted as a ratio of nongerminated conidia compared to the negative control. The experiment was performed in duplicate. The results are summarized in Table 4.

#### 3.3.9. In Vitro Antiproliferative Assay

Human monocytic leukemia THP-1 cells were used for in vitro antiproliferative assay. Cells were obtained from the European Collection of Cell Cultures (ECACC, Salisbury, UK) and routinely cultured in RPMI (Roswell Park Memorial Institute) 1640 medium supplemented with 10% fetal bovine serum, 2% l-glutamine, 1% penicillin, and streptomycin at 37 °C with 5% CO_2_. Cells were passaged at approximately one-week intervals. The antiproliferative activity of the compounds was determined using a Water Soluble Tetrazolium Salts-1 (WST-1, 2-(4-iodophenyl)-3-(4-nitrophenyl)-5-(2,4-disulfophenyl)-2*H*-tetrazolium) assay kit (Roche Diagnostics, Mannheim, Germany) according to the manufacturer’s instructions. The tested compounds were dissolved in DMSO and added in five increasing concentrations (0.37, 1.1, 3.3, 10, and 30 μM) to the cell suspension in the culture RPMI 1640 medium. The maximum concentration of DMSO in the assays never exceeded 0.1%. Subsequently, the cells were incubated at 37 °C with 5% CO_2_ for 24 h. For WST-1 assays, cells were seeded into 96-well plates (5 × 10^4^ cells/well in 100 μL culture medium) in triplicate in serum-free RPMI 1640 medium, and measurements were taken 24 h after the treatment with the compounds. The median inhibition concentration values, IC_50_, were deduced through the production of a dose-response curve. All data were evaluated using GraphPad Prism 5.00 software (GraphPad Software, San Diego, CA, USA). The results are shown in Table 2.

#### 3.3.10. Study of Inhibition of Photosynthetic Electron Transport (PET) in Spinach Chloroplasts

Chloroplasts were prepared from spinach (*Spinacia oleracea* L.) according to Masarovicova and Kralova [94]. The inhibition of photosynthetic electron transport (PET) in spinach chloroplasts was determined spectrophotometrically (Genesys 6, Thermo Scientific), using an artificial electron acceptor 2,6-dichlorophenol-indophenol (DCIPP) according to Kralova et al. [95], and the rate of photosynthetic electron transport was monitored as a photoreduction of DCPIP. The measurements were carried out in phosphate buffer (0.02 mol/L, pH 7.2) containing sucrose (0.4 mol/L), MgCl_2_ (0.005 mol/L), and NaCl (0.015 mol/L). The chlorophyll content was 30 mg/L in these experiments, and the samples were irradiated (~100 W/m^2^ with 10 cm distance) with a halogen lamp (250 W) using a 4 cm water filter to prevent warming of the samples (suspension temperature 22 °C). The studied compounds were dissolved in DMSO due to their limited water solubility. The applied DMSO concentration (up to 4%) did not affect the photochemical activity in spinach chloroplasts. The inhibitory efficiency of the studied compounds was expressed by IC_50_ values, i.e., by molar concentration of the compounds causing a 50% decrease in the oxygen evolution rate relative to the untreated control. The comparable IC_50_ value for the selective herbicide 3-(3,4-dichlorophenyl)-1,1-dimethylurea, DCMU (Diuron^®^) was about 2.1 μmol/L. The results are shown in Table 2.

#### 3.3.11. In Vivo Toxicity against Plant Cells

*Nicotiana tabacum* var. Samsun was used for this test. The compounds were diluted in water to reach concentrations equal to 1× MIC and 4× MIC. As the negative control, solutions of DMSO in the same concentration were used. The positive control was 5% DMSO, which should be toxic to the plant cells. The aqueous solutions were injected into the plant leaves by a syringe (needle 27G × ¾”) to fill area approx. 1 cm^2^ [88]. The test was made in triplicates in three different plants. The plants were kept in a greenhouse on direct sunlight. The effect of the compounds was visually checked every day for 5 weeks. In the presence of tested compounds, no visible changes in the plant tissues were observed. The results are illustrated in Figure 6.

## 4. Conclusions

A series of sixteen ring-substituted *N*-arylcinnamamides was prepared, characterized, and evaluated against *Staphylococcus aureus*, three methicillin-resistant *S. aureus* strains, *Mycobacterium tuberculosis* H37Ra, *Fusarium avenaceum*, and *Bipolaris sorokiniana*. Additionally, the compounds were tested for their activity related to the inhibition of photosynthetic electron transport in spinach chloroplasts. (2*E*)-3-Phenyl-*N*-[3-(trifluoromethyl)phenyl]prop-2-enamide (**6**) and (2*E*)-*N*-[3,5-bis(tri-fluoromethyl)phenyl]-3-phenylprop-2-enamide (**13**) showed the highest activity (MIC = 27.47 and 22.27 µM, respectively) against all four staphylococcal strains as well as against *M. tuberculosis*. These compounds also showed activity against bacterial biofilm forming, the ability to increase the effect of clinically used antibiotics such as tetracycline, vancomycin, and ciprofloxacin and concentration-dependent antibacterial effect, which led to killing >99% of bacteria. On the other hand, (2*E*)-*N*-(3-fluorophenyl)- (**5**) and (2*E*)-*N*-(3-methylphenyl)-3-phenylprop-2-enamide (**2**) had MICs = 16.58 and 33.71 µM, respectively, against *B. sorokiniana*, while both compounds did not show any in vivo toxicity against *Nicotiana tabacum* var. Samsun. (2*E*)-*N*-(3,5-dichlorophenyl)-3-phenylprop-2-enamide (**11**, IC_50_ = 5.1 µM) was the most active PET inhibitor. These compounds showed activities comparable with or higher than those of standards. A significant decrease of mycobacterial cell metabolism (viability of *M. tuberculosis* H37Ra) was observed using the MTT assay. The screening of the cytotoxicity of the selected compounds was performed using THP-1 cells, and no significant lethal effect was observed for the most potent compounds. The position of substituents on the anilide ring seems to be crucial for both antitubercular and antifungal activity; positions C_(3)_’, C_(3,4)_’, and C_(3,5)_’ are preferable. Lipophilicity is another important factor; antitubercular activity increases with increasing lipophilicity, while antifungal activity, to the contrary, decreases from the most effective compound **5** (R = 3-F) with lipophilicity log *k* = 0.23 with increasing lipophilicity. The activity is also dependent on the electronic parameters of substituents on the anilide core. Bilinear trends for effective compounds can be observed for both antitubercular and antifungal dependences; nevertheless, for antitubercular effectivity, rather more electron-withdrawing properties are preferred (σ_Ar_ ≈ 1, compound **13**, R = 3,5-CF_3_), while for antifungal activity against *B. sorokiniana*, less electron-withdrawing properties are preferred (σ_Ar_ = 0.82, compound **5**, R = 3-F). Also the dependences of PET-inhibiting activity on lipophilicity and electronic properties showed bilinear trends, as mentioned above, where C_(3,5)_’ or C_(3,4)_’ substitution of the anilide core is preferable.

## Figures and Tables

**Table 1:**
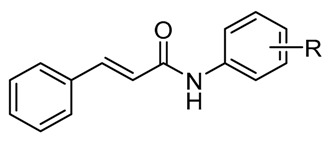
Structure of ring-substituted (2*E*)-*N*-aryl-3-phenylprop-2-enamides **1**–**16**, experimentally determined values of lipophilicity log *k*, calculated values of log *P*/Clog *P*, and electronic Hammett’s σ parameters.

Comp.	R	log *k*	Clog *P ^a^*	log *P ^b^*	σ_Ar_ *^b^*
**1**	H	0.1146	3.6640	3.18	0.60
**2**	3-CH_3_	0.2729	4.1630	3.40	0.48
**3**	4-CH_3_	0.2640	4.1630	3.40	0.46
**4**	2-F	0.1330	3.4646	3.17	1.02
**5**	3-F	0.2327	4.0646	3.32	0.82
**6**	3-CF_3_	0.4859	4.9978	4.26	0.89
**7**	2,5-CH_3_	0.2691	4.0120	3.57	0.59
**8**	2,5-Cl	0.5799	4.5878	4.65	1.22
**9**	2,6-Cl	0.0632	3.7378	4.56	1.33
**10**	3,4-Cl	0.6821	5.3178	4.70	1.19
**11**	3,5-Cl	0.8155	5.4378	4.79	1.11
**12**	2,6-Br	0.0992	3.9778	4.80	1.33
**13**	3,5-CF_3_	0.9814	6.0386	5.68	1.05
**14**	2-F-5-Br	0.4875	4.4178	4.07	1.28
**15**	2-Br-5-F	0.4588	4.1378	4.12	1.19
**16**	2-Cl-5-CF_3_	0.6178	4.9509	4.88	1.19

*^a^* calculated using ChemBioDraw Ultra 13.0; *^b^*calculated using ACD/Percepta ver. 2012.

**Table 2:** Structure of ring-substituted (2*E*)-*N*-aryl-3-phenylprop-2-enamides **1**–**16**, IC_50_ (μM) values related to PET inhibition in spinach chloroplasts in comparison with 3-(3,4-dichlorophenyl)-1,1-dimethylurea (DCMU) standard, in vitro anti-*Staphylococcus* activities MIC (μM) in comparison with standard ampicillin (AMP), in vitro antitubercular activity MIC (μM (µg/mL)) in comparison with standard isoniazid (INH), in vitro antifungal activity MIC (μM (µg/mL)) of compounds **1**–**16** compared to standard benomyl (BNM), and in vitro antiproliferative (Tox) assay (IC_50_ (μM)) of chosen compounds compared to standard camptothecin (CMP).

Comp.	R	MIC (μM (µg/mL))							Tox IC_50_ (μM)	PET IC_50_ (μM)
		SA	MRSA 63718	MRSA SA 630	MRSA SA 3202	Mtb	FA	BS		
**1**	H	>1146 (>256)	>1146 (>256)	>1146 (>256)	>1146 (>256)	286 (64)	1146 (256)	143 (32)	–	250
**2**	3-CH_3_	>1078 (>256)	>1078 (>256)	>1078 (>256)	>1078 (>256)	67.43 (16)	270 (64)	33.71 (8)	>30	343
**3**	4-CH_3_	>1078 (>256)	>1078 (>256)	>1078 (>256)	>1078 (>256)	134 (32)	1078 (256)	539 (128)	–	320
**4**	2-F	>1061 (>256)	>1061 (>256)	>1061 (>256)	>1061 (>256)	265 (64)	1061 (256)	66.32 (16)	–	223
**5**	3-F	>1061 (>256)	>1061 (>256)	>1061 (>256)	>1061 (>256)	66.31 (16)	531 (128)	16.58 (4)	>30	165
**6**	3-CF_3_	27.47 (8)	27.47 (8)	27.47 (8)	27.47 (8)	27.47 (8)	54.93 (16)	54.93 (16)	22.72 ± 1.73	189
**7**	2,5-CH_3_	>1018 (>256)	>1018 (>256)	>1018 (>256)	>1018 (>256)	254 (64)	1019 (256)	1019 (256)	–	338
**8**	2,5-Cl	>876 (>256)	>876 (>256)	>876 (>256)	>876 (>256)	876 (256)	876 (256)	876 (256)	–	67.1
**9**	2,6-Cl	>876 (>256)	>876 (>256)	>876 (>256)	>876 (>256)	876 (256)	876 (256)	876 (256)	–	1380
**10**	3,4-Cl	438 (128)	876 (256)	438 (128)	876 (256)	27.38 (8)	219 (64)	110 (32)	29.81 ± 0.31	54.9
**11**	3,5-Cl	438 (128)	876 (256)	109 (64)	438 (128)	27.38 (8)	219 (64)	110 (32)	29.44 ± 1.73	5.1
**12**	2,6-Br	>671 (256)	>671 (256)	>671 (256)	>671 (256)	167 (64)	671 (256)	671 (256)	–	732
**13**	3,5-CF_3_	22.27 (8)	22.27 (8)	22.27 (8)	22.27 (8)	22.27 (8))	713 (256)	356 (128)	22.59 ± 1.88	111
**14**	2-F-5-Br	>799 (>256)	>799 (>256)	>799 (>256)	>799 (>256)	199 (64)	799 (256)	49.98 (16)	–	188
**15**	2-Br-5-F	>799 (>256)	>799 (>256)	>799 (>256)	>799 (>256)	199 (64)	799 (256)	799 (256)	–	205
**16**	2-Cl-5-CF_3_	>785 (>256)	>785 (>256)	>785 (>256)	>785 (>256)	785 (256)	785 (256)	785 (256)	–	63.2
**AMP**	–	5.72 (2)	45.81 (16)	45.81 (16)	45.81 (16)	–	–	–	–	–
**INH**	–	–	–	–	–	36.55 (5)	–	–	–	–
**BNM**	–	–	–	–	–	–	1.94 (0.5)	17.22 (5)	–	–
**CMP**	–	–	–	–	–	–	–	–	0.16 ± 0.07	–
**DCMU**	–	–	–	–	–	–	–	–	–	2.1

SA *= Staphylococcus aureus* ATCC 29213; MRSA *=* clinical isolates of methicillin-resistant *S. aureus* 63718, SA 630, and SA 3202 (National Institute of Public Health, Prague, Czech Republic); Mtb = *Mycobacterium tuberculosis* H37Ra; FA = *Fusarium avenaceum* (Fr.) Sacc. IMI 319947; BS = *Bipolaris sorokiniana* (Sacc.) Shoemaker H-299 (NCBI GenBank accession No. MH697869).

**Table 3:** Combined effect of most potent *N*-arylcinnamamides and tetracycline (TET), ciprofloxacin (CPX), and vancomycin (VAN).

Isolate	Combination of Compds.	Separate MIC (μg/mL)	FIC Index	Concentration (μg/mL) Causing Synergistic Effect	Concentration (μg/mL) Causing Additive Effect
MRSA 63718	**6**/TET	8/128	1.004–2.250	–	2/64; 8/32
	**6**/CPX	16/16	0.75–1.125	–	8/4; 4/8
	**6**/VAN	32/2	1.000–1.250	–	–
MRSA SA 3202	**6**/TET	16/64	1.002–1.25	–	–
	**6**/CPX	8/8	1.000–1.250	–	–
	**6**/VAN	8/1	0.750–1.256	–	4/0.25
	**13**/TET	32/64	0.500–1.125	8/16	16/16; 4/32; 2/64
	**13**/CPX	32/8	0.375–1.250	8/1	2/4
	**13**/VAN	32/1	0.750–1.25	–	16/0.25
MRSA SA 630	**6**/CPX	8/256	0.625–1.125	–	4/64; 1/128
	**6**/VAN	8/1	0.750–1.250	–	2/0.5
	**13**/CPX	8/256	0.375–1.004	2/32; 1/64	4/8
	**13**/VAN	4/1	0.562–1.250	–	0.25/0.5

**Table 4:** Inhibition (%) of *Bipolaris sorokiniana* conidium germination by compounds **1**–**16** in comparison to negative control. Benomyl (BNM) was used as positive control.

Comp.	Concentration (μg/mL)	Inhibition (%) Compared to Negative Control	Comp.	Concentration (μg/mL)	Inhibition (%) Compared to Negative Control
**1**	256	59.6	**9**	256	82.5
	128	74.2		128	76.3
**2**	256	64.7	**10**	256	84.3
	128	59.1		128	51.3
**3**	256	88.7	**11**	256	84.8
	128	89.1		128	82.6
**4**	256	60.8	**12**	256	91.3
	128	58.7		128	82.1
**5**	256	93.6	**13**	256	76.3
	128	95.4		128	75.5
**6**	256	18.4	**14**	256	81.8
	128	26.7		128	77.6
**7**	256	73.5	**15**	256	100
	128	35.1		128	60.9
**8**	256	92.7	**16**	256	86.2
	128	88.7		128	61.2
BNM	10	100	BNM	10	100
	5	100		5	100
